# Development of Content for a Virtual Reality Simulation to Understand and Mitigate Moral Distress in Healthcare Workers

**DOI:** 10.7759/cureus.31240

**Published:** 2022-11-08

**Authors:** Mithusa Sivanathan, Caroline W Espinola, Alvaro Uribe Quevedo, Bill Kapralos, Sridhar Krishnan, Venkat Bhat, Adam Dubrowski

**Affiliations:** 1 Health Sciences, Ontario Tech University, Oshawa, CAN; 2 Psychiatry, St. Michael’s Hospital, Toronto, CAN; 3 Software and Informatics Research Centre, Ontario Tech University, Oshawa, CAN; 4 Electrical, Computer, and Biomedical Engineering, Toronto Metropolitan University, Toronto, CAN

**Keywords:** mental health, psychiatry, psychology, educational resource, virtual reality, healthcare simulation, moral injury, moral distress, mobile app, covid-19

## Abstract

Background

In high-stakes situations, healthcare workers are prone to suffer moral injury, the psychological, social, and spiritual impact of events involving betrayal or transgression of one’s own deeply held moral beliefs and values. As a result, this may negatively impact their capacity to provide adequate levels of care to patients. There is a lack of educational resources catered to help healthcare workers navigate ethical situations in clinical settings that may lead to or worsen moral distress. The aim of this report is to describe the methodology of development and resulting outcomes in the form of an educational resource that includes a virtual reality (VR) simulation to help healthcare workers understand and mitigate moral distress as a result of internal and external constraints at their workplaces.

Methodology

A study using a method outlining a set of constraint parameters, followed by ideation utilizing design thinking (DT), and concluding with a consensus-building exercise using Delphi methodology (DM) with a group of 13 experts in healthcare simulation, VR, psychiatry, psychology, and nursing. The constraints parameters included technology use (VR), use of experiential learning theory, and duration of the intervention (15 minutes). A DT process was performed to generate and expand on ideas on the scenario and intervention of a possible VR simulation which were funneled into a three-round DM to define the foundations of the VR simulation. Average, standard deviations, and free-text comments in the DM were used to assess the inclusion of the produced requirements. Finally, a focus group interview was conducted with the same experts to draft the VR simulation.

Results

Within the specified constraints, the DT process produced 33 ideas for the VR simulation scenario and intervention that served as a starting point to short-list the requirements in Round 1. In Rounds 1 to 2, 25 items were removed, needed revising, and/or were retained for the subsequent rounds, which resulted in eight items at the end of Round 2. Round 2 also required specialists to provide descriptions of potential scenarios and interventions, in which five were submitted. In Round 3, experts rated the descriptions as somewhat candidate to use in the final VR simulation, and the open feedback in this round proposed combining the elements from each of the descriptions. Using this data, a prototype of the VR simulation was developed by the project team together with VR designers.

Conclusions

This development demonstrated the feasibility of using the constraints-ideation-consensus approach to define the content of a possible VR simulation to serve as an educational resource for healthcare workers on how to understand and mitigate moral distress in the workplace. The methodology described in this development may be applied to the design of simulation training for other skills, thereby advancing healthcare training and the quality of care delivered to the greater society.

## Introduction

Internal and external constraints in clinical settings have caused healthcare providers to transgress on deeply held moral values and commitments [1]. When this occurs, their moral foundation is threatened or violated which can lead to moral distress [2], negatively impacting their emotional and physical well-being [3,4], which, in turn, can diminish their capacity to provide adequate levels of care to patients [5].

To date, there is limited research on factors leading to and the treatment of moral distress in healthcare providers as the concept of moral distress is not diagnostically coded and remains ill-defined [6,7]. Gilligan (2014) suggested that healthcare providers who are chronically exposed to moral distress may suffer from subsequent moral injury, “a shattering of trust that compromise[s] our ability to love” [8]. Because moral injury and post-traumatic stress disorder (PTSD) have overlapping symptoms, treatments targeting PTSD symptomatology have been used to target moral injury. However, this has generally rendered them unresponsive [9]. Fortunately, healthcare providers’ moral compass can be repaired by strengthening it through healthcare ethical education. Then, they may be ready to handle ethical challenges and the effects of moral distress which can lead to better patient outcomes [10-12]. Currently, there is a lack of sufficient educational resources to help healthcare providers navigate ethically challenging situations [13].

To address this gap, we propose to develop and test an educational resource that is rooted in Kolb’s experiential learning theory and supported by a digital technology platform consisting of virtual reality (VR) simulation to understand and mitigate the effects of moral distress on healthcare workers. This original article aims to (a) describe the use of a novel, hybrid methodology, which sets design constraints and combines elements of two consensus-building methodologies, a design thinking (DT) process and Delphi methodology (DM), with an interdisciplinary team to define and scope the parameters, needs, and requirements to develop content for the VR simulation, and (b) to describe the final outcome of this development.

## Materials and methods

As of March 20, 2021, the research ethics board at St. Michael’s Hospital, Unity Health Toronto has approved the study, with the reference number UHTDTS25377.

Participants 

The interdisciplinary team was a group of 13 individuals. The following disciplines were represented: five in psychiatry, three in psychology, three in nursing, one in game development, and one in healthcare simulation.

The project team consisted of eight individuals who prepared and facilitated the development process. There was one engineer, two computer scientists, one healthcare simulation specialist, one healthcare simulation researcher, two psychiatrists, and one health sciences graduate student.

Design constraints

There were three design constraints that were imposed on the development of the simulation, namely, learning theory, technology, and time. Kolb’s experiential learning theory was selected as an educational framework to structure the educational resource. Kolb’s experiential learning theory consists of the following four stages: concrete experience, reflection, conceptualization, and active experimentation. Following this cycle, an individual learns effectively when they have had (1) exposure to a real-life event, (2) followed by observation and reflection on that experience. This then leads to (3) the formation of abstract concepts and generalizations, (4) which are then used to test a hypothesis by applying what they have learned in future situations, resulting in new experiences [14].

**Figure 1:**
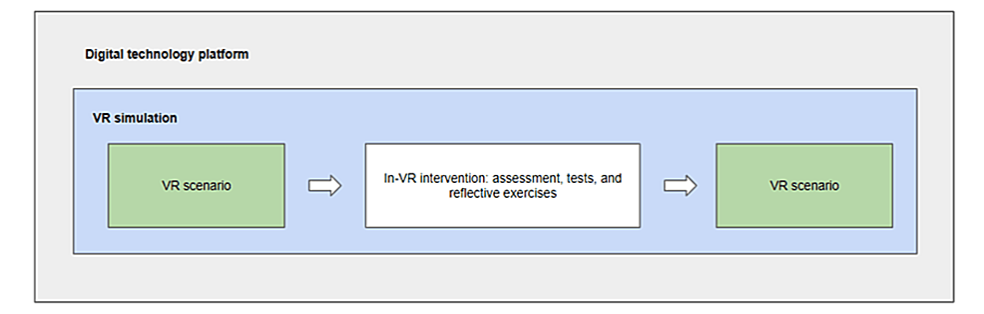
The educational resource consists of a digital technology platform which is made up of a VR simulation. The VR simulation is structured according to Kolb’s experiential learning theory and takes the learner through a VR scenario, an in-VR intervention, and then the VR scenario again. VR: virtual reality

In this conception, as it is logistically and ethically unacceptable to expose healthcare providers to real-life events that evoke moral distress, VR will provide these experiential opportunities, hence, the selection of this technology. Specifically, concrete experiences will be provided to healthcare workers by immersing them in the VR simulation that will trigger moral distress. Reflection and conceptualization will be facilitated by an in-VR intervention made up of assessments, tests, and reflective exercises, which will be used to guide the learner to reflect on the experience, providing them with knowledge and skills related to dealing with moral distress in the future. The in-VR intervention will be based on a concept known as “psychological first aid” from “A guide to moral injury” [15]. This guide offers advice on how to implement preventative and early intervention structures to support healthcare providers before they are morally injured and outlines the support that needs to be provided at the organizational, team, and individual levels. Finally, active experimentation will be achieved by having the healthcare participant undergo the same VR simulation again so they can apply and test the newly learned concepts from the in-VR intervention (Figure 1).

This VR simulation is intended to be structured as a 10-minute scenario, a 10-minute in-VR intervention, and then the VR simulation again, totaling 30 minutes. The intent for this length is to provide enough content to participants, which includes the experience and teachings, during the VR simulation while keeping it short enough to encourage recruitment and capture emotional reactions from participants as they occur.

Hybrid design thinking and Delphi methodology

DT is an iterative and non-linear process that provides a solution-based approach to solving complex problems. It comprises the following five stages: (1) empathizing - understanding the human needs involved; (2) defining - re-framing and defining the problem in human-centric ways; (3) ideating - brainstorming as many ideas in ideation sessions; (4) prototyping - adopting a hands-on approach in prototyping; and (5) testing - developing a prototype/solution to the problem. It is useful in tackling complex problems that are ill-defined or unknown and within a multidisciplinary team setting where the diversity of thinking accelerates the process [16].

**Figure 2:**
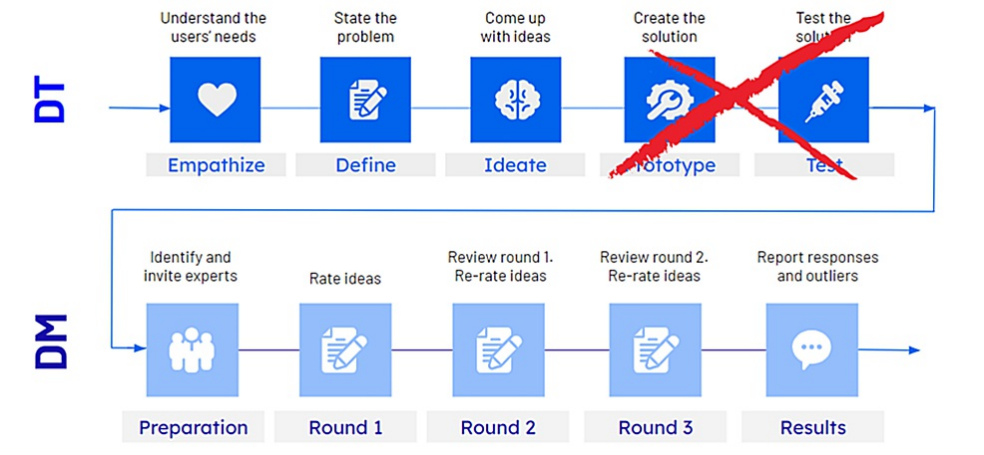
The DT-DM is a novel consensus-building methodology that is a combination of DT and DM elements. The first three phases of DT, empathize, define, and ideate, are involved to help generate and expand on ideas. Before prototyping and testing the ideas, the ideas need evidence of content validity to be accepted by the medical research community. To achieve this, the ideas are put through a DM in which the ideas are fine-tuned until an expert consensus is reached. DT: design thinking; DM: Delphi methodology

For the educational resource, we chose to focus on only the first three stages of the DT process with an interdisciplinary team to generate a set of ideas that were discussed and classified based on their feasibility as promising and valid. These valid and promising ideas required content validity, a measure of how well the content of the VR simulation reflects real-life situations and learning needs [17]. To accomplish this, we skipped the last two stages of the DT process, namely, prototype and test, and instead, moved the valid and promising ideas into a three-round DM (Figure 2). Due to the elimination of DT steps in the process, the DT in this original article will be considered modified.

The DM is a group communication process that seeks to gather information and achieve consensus from a panel of experts using iterative survey questionnaires [18]. It is widely used in medical education and has been applied in needs assessment processes [19], in identifying content for assessment instruments [20], and in research priorities in medical education [21].

The first survey questionnaire for the DM was formulated by the project team by taking all valid and promising ideas from the modified DT and structuring each of them into a question that asked to rank their importance. Because these questions were pre-defined by the DT in the DM which is different from the conventional DM where the starting questions are open-ended, the DM will be referred to as modified [22]. The same interdisciplinary team that took part in the DT was given three rounds of the DM survey questionnaires to gather their insights on the topic and come to an agreement on the criteria for the content of the VR simulation.

Focus group interview

The output from Round 3 of the DM was then used to draft the content in the form of a script for the VR simulation scenario and a slide deck for the in-VR intervention. The script and slide deck drafts were shared via Google Docs and Google Slides, respectively, with the 13 participants for their feedback for a week. The comments for both the script and slide deck drafts were integrated accordingly. These preliminary drafts were then discussed during a one-hour Zoom meeting focus group interview with the same 13 participants to address any outstanding comments. The revised script and slide deck drafts were then provided to two VR designers to create a prototype of the VR simulation. The first VR simulation prototype was created in two weeks, and a video recording of the prototype was then given to the participants for their feedback.

Constraints-ideation-consensus approach

**Figure 3:**
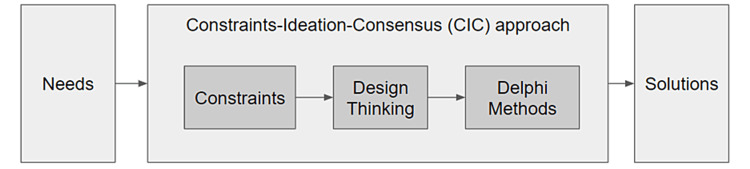
The constraints-ideation-consensus approach.

In summary, the process of creating content for the VR simulation underwent the following steps: (1) the identification of constraints, (2) the generation of ideas through DT, (3) a DM using the DT ideas for expert consensus, and (4) a focus group interview to clarify the outcome of the DM. This sequence of tools will be referred to as the constraints-ideation-consensus (CIC) approach (Figure 3). Participants who took part in the CIC approach were sent a Google Form survey in the form of a SWOT analysis to provide feedback on the methodology which inquires about the strengths, weaknesses, opportunities, and threats of the process [23].

Protocol

Modified Design Thinking

The modified DT process took place over a two-hour Zoom meeting with 13 participants. To ensure ideas for content were gathered for all parts of the VR simulation according to Kolb’s experiential learning theory, the VR scenario and the in-VR intervention, participants were then assigned to one of the three areas, namely, scenario, intervention, and assessments, through the use of breakout rooms in Zoom. For each breakout room, a facilitator guided the participants through the first three stages of the modified DT process and recorded all ideas for each stage on a Google Jamboard. After the modified DT process, the ideas were summarized and integrated into the modified DM.

Modified Delphi Methodology

Afterward, a modified DM was conducted with the same 13 participants who took part in the modified DT process. The modified DM took the form of a survey questionnaire hosted in Google Forms and was sent electronically to the 13 participants to complete via email within one week per round. The survey questionnaires were based on the ideas generated from the modified DT process. In each round, participants were asked to rate items to include in the content of the VR simulation on a Likert scale of one to five, one being very unimportant and five being very important [24]. For each item, participants were provided the opportunity to provide comments through a free-text field. After each round, the results were statistically analyzed using mean and standard deviation (SD). Items with a mean of 3.5 or greater and demonstrated low variability (i.e., SD less than 1) were considered important by the majority of participants. These items were noted as elements to include in the final content of the VR simulation and were not revisited in the subsequent rounds of the modified DM. Items that had a mean lower than 3.5 and demonstrated low variability (i.e., SD less than 1) were considered unimportant by the majority of participants. These items were noted as elements to not include in the final content of the VR simulation and were not revisited in the subsequent rounds of the DM. Items that displayed high variability (i.e., SD equal to 1 or greater) suggested a lack of consensus among participants on the inclusion of the element in the final content of the VR simulation [24]. To gain clarity on these particular items along with any of their comments in the associated free-text fields, after the relevant modified DM round was concluded, a one-hour Zoom meeting was conducted with the participants. Then, these items were revisited in the subsequent rounds of the modified DM through the survey questionnaires until an agreement on the inclusion of the items among the participants was achieved. The modified DM process garnered consensus among the participants by the third round and this took approximately three weeks to complete, given one week per round.

## Results

Modified design thinking

During the modified DT process, 13 participants generated 33 valid and promising ideas for the content of the VR simulation (Table 1). The ideas served as the items for the survey questionnaires for the modified DM Round 1 and free-text fields were included to gather any comments, questions, and suggestions on the overall VR simulation (Table 2).

**Table 1:** List of 33 valid and promising ideas generated from the modified DT process. VR: virtual reality; DT: design thinking

VR Simulation Scenario Ideas
Virtual actors doing self-harm
Virtual actors being dehydrated
Have virtual actors lacking sleep
Have a moral debate between virtual actors (scripted) and the participant observes the interaction
Have virtual actors experiencing misplaced anger
Have the participant observe interactions between virtual actors
Have virtual actors being exposed to a demonstration of bad leadership
Have virtual actors making mistakes unintentionally/intentionally
Offer a team experience
Require that the participant goes through a decision-making tree
Make the participant aware that they are in a moral dilemma
Have virtual actors feel unsafe
Require the participant to make time-sensitive decisions
Have virtual actors experiencing frustration
Have virtual actors feeling betrayed
Offer an individual experience
Have the participant actively interact with virtual actors
Have virtual actors feeling the effects of staffing issues
Include evocative storytelling
Trigger emotional responses in the participant
Prompt reflection in the participant
Shows something has happened and the participant goes through the experience
Elicit genuine reactions from the participant which drives their responses to the intervention
in-VR Simulation Intervention Ideas
Have the participant identify the values in conflict
Have a moral debate between virtual actors and the participant (scripted)
Have various pathways that can help the participants navigate the scenario
Teach the participant about moral distress
Require the participant to experience moral distress
in-VR Simulation Assessment Ideas
Use participant biometric data (e.g., physiological markers of stress levels) gathered during the VR simulation to request participants to share reflections on the experience and suggest tools provided during the “intervention” that would help deal them with this situation in the future
Require the participant to complete “perceptual scales” (e.g., anxiety scales/stress index) such as pop-up in-scenario quizzes (provide results concurrently) based on standardized instruments
Require the participant to complete “reflective” short writing tasks (asks the participant to provide a summary as terminal feedback) based on standardized instruments
Require the participant to complete matching “logic” games based on standardized instruments
Include in-scenario metrics of performance that would provide feedback (concurrent) about logic/reasoning or correctness (if applicable)

**Table 2:** List of questions for modified DM Round 1. VR: virtual reality; DM: Delphi methodology; ICU: intensive care unit; ER: emergency room

Questions #	Modified DM Round 1 question
1	How important is it that the VR scenario is developed to: Have virtual actors doing self-harm?
2	How important is it that the VR scenario is developed to: Have virtual actors being dehydrated?
3	How important is it that the VR assessments are developed to: Require the participant to complete matching “logic” games based on standardized instruments?
4	How important is it that the VR assessments are developed to: Include in-scenario metrics of performance that would provide feedback (concurrent) about logic/reasoning or correctness (if applicable)?
5	How important is it that the VR scenario is developed to: Have virtual actors lacking sleep?
6	How important is it that the intervention is developed to: Have a moral debate between virtual actors (scripted) and the participant observes the interaction?
7	How important is it that the VR assessments are developed to: Require the participant to complete “reflective” short writing tasks (ask the participant to provide a summary as terminal feedback) based on standardized instruments?
8	How important is it that the VR scenario is developed to: Have virtual actors experiencing misplaced anger?
9	How important is it that the intervention is developed to: Have the participant identify the values in conflict?
10	How important is it that the intervention is developed to: Have a moral debate between virtual actors and the participant (scripted)?
11	How important is it that the VR scenario is developed to: Have the participant observe interactions between virtual actors?
12	How important is it that the VR scenario is developed to: Have virtual actors exposed to a demonstration of bad leadership?
13	How important is it that the intervention is developed to: Have various pathways that can help the participants navigate the scenario?
14	How important is it that the intervention is developed to: Teach the participant about moral distress? This was the emphasis in Phase 1, see the video above.
15	How important is it that the intervention is developed to: Require the participant to experience moral distress?
16	How important is it that the VR assessments are developed to: Require the participant to complete “perceptual scales” (e.g., anxiety scales/stress index) such as pop-up in-scenario quizzes (provide results concurrently) based on standardized instruments?
17	How important is it that the VR scenario is developed to: Have virtual actors making mistakes unintentionally/intentionally?
18	How important is it that the VR scenario is developed to: Offer a team experience?
19	How important is it that the VR scenario is developed to: Require that the participant goes through a decision-making tree?
20	How important is it that the intervention is developed to: Make the participant aware that they are in a moral dilemma?
21	How important is it that the VR scenario is developed to: Have virtual actors feeling unsafe?
22	How important is it that the intervention is developed to: Require the participant to make time-sensitive decisions?
23	How important is it that the VR scenario is developed to: Have virtual actors experiencing frustration?
24	How important is it that the VR scenario is developed to: Have virtual actors feeling betrayed?
25	How important is it that the VR scenario is developed to: Offer an individual experience?
26	How important is it that the VR scenario is developed to: Have the participant actively interacting with virtual actors?
27	How important is it that the VR scenario is developed to: Have virtual actors feeling the effects of staffing issues?
28	How important is it that the VR scenario is developed to: Include evocative storytelling?
29	How important is it that the VR scenario is developed to: Trigger emotional responses in the participant?
30	How important is it that the VR assessments are developed to: Use participant biometric data (e.g., physiological markers of stress levels) gathered during the VR simulation to request participants to share reflections on the experience and suggest tools provided during the “intervention” that would help deal them this situation in the future?
31	How important is it that the VR scenario is developed to: Prompt reflection in the participant?
32	How important is it that the VR scenario is developed to: Show something has happened and the participant goes through the experience?
33	How important is it that the VR scenario is developed to: Elicit genuine reactions from the participant which drives their responses to the intervention?
34	Please provide an optimal setting for this scenario (e.g., ICU, ER, Long-term care, outpatient). It would be beneficial to have a scenario that any nursing professional could relate to as a specialized scenario (e.g., ICU) will exclude nursing professionals working in other settings and not allow for the recruitment of 100 participants over 6 months.
35	Please provide any comments/suggestions/questions for the OVERALL development of the VR assessments.
36	Please provide any comments/suggestions/questions for the OVERALL development of the VR intervention.
37	Please provide any comments/suggestions/questions for the OVERALL development of the VR scenario.
38	Please provide any comments/suggestions/questions for the OVERALL development of the VR simulation.

Modified Delphi methodology

In Round 1 of the modified DM, all 13 participants rated the importance of the inclusion of the 33 items from the modified DT process in a survey questionnaire. Statistical analysis and review of free-text field commentaries provided in Round 1 led to nine items being removed, 12 items being retained, and 12 items requiring further clarification from the participants (Table 3). After Round 1 ended, a Zoom meeting with the participants resulted in the 12 items merging into eight items requiring revisiting in Round 2 (Table 4).

**Table 3:** List of modified DM Round 1 items/sketches, free-text field comments, means, and SDs. * Item originally “Revisit for Round 2” based on statistical analysis, however, was reclassified based on comments/end-of-round meeting discussions. SD: standard deviation; ICU: intensive care unit; MI: moral injury; DM: Delphi methodology; COVID-19: coronavirus disease 2019; PTSD: post-traumatic stress disorder

Question #	Average	SD	Decision for modified DM Round 1	Comments
1	2.08	1.00	Remove	Way too low a base rate
				There should be harm, but not likely self-harm
				Typically a very rare event in a real scenario
				Too extreme
				Stopping self-harm likely not a dilemma for a nurse
2	2.33	0.78	Remove	Dehydrated? Excess alcohol consumption?
				In a war zone maybe not here
3	2.75	1.06	Remove*	What is this and what is the aim?
				Logic? This sounds like the early days of bioethics when it was believed that ethical theory would make our ethical problems go away. Logic can be good for dilemmas but not so much for moral distress.
4	3.00	1.13	Remove*	What is this and what is the aim?
				Again, the reasoning isn’t the problem otherwise all the years of ethics education in nursing would have reduced moral distress
				Development of models that would assist in predicting and responding to risks associated with “moral injury” during the next phase of the pandemic or in the future, including, but not limited to development of critical models of disease prevention and psychological, neurological, and endocrine intervention models for responding to risks
				Must balance this to make sure we aren’t interfering with the process
5	3.08	1.16	Remove*	It might help the scenario, but isn’t necessary
				Sleep is important obviously but not central to moral distress.
				Sleep loss, maladaptive coping, and adverse outcomes are important
6	3.17	1.34	Remove*	What is a moral debate and what would that achieve?
				This is a good way to get at the concept of moral distress
				This is a core requirement for decision making so important to include this
				It would be good if perhaps someone were taking a position that is unethical.
				Post-funeral reflection with a peer (“root-cause analysis”) could be considered, what was the nurse going through (emotions & thoughts) and decision-making framework debate either internal or external is important
7	3.17	1.27	Remove*	Too clunky
				Would be nice, but not necessary
				I think we will get more out of them by asking them to speak as opposed to write
				Could this be the debrief?
				Want to avoid burden but a qualitative way to see if we hit the mark
8	3.25	1.14	Revisit for Round 2	This all depends if we are trying to evoke other-directed MI or self-directed MI
				Why misplaced? When is anger misplaced?
				This may well trigger HCP, we’ll have to be able to manage this
				Anger isn’t always part of moral distress
				This is possible in a natural scenario so good to include that component
				It's hard to know where to place anger when you don't have much power. Where would it be properly placed? Political action may be...
				Anger turned inwards!-maladaptive behavior by the nurse-poor team/organizational response
				Anger good not sure we can determine misplaced
9	3.42	1.51	Revisit for Round 2	The types of conflicts we may expose them to may not be productive
				The values might not be in conflict--because again that’s more of a dilemma. It is important for participants to be able to express what values/beliefs etc are being compromised. It’s important in any scenario that participants be able to express themselves using ordinary language. The “values” can be identified if necessary by someone doing the data analysis.
				Values in conflict and maladaptive responses
10	3.50	1.24	Remove*	Don’t get it
				Helps with engagement
				Great to have the first-person focus--but the scripted element limits what is possible
				Post-funeral reflection with a peer (“root-cause analysis”) could be considered, what was the nurse going through (emotions & thoughts) and decision-making framework
				Debate with internal actors can accentuate it
11	3.50	1.17	Remove*	I think it is more important that the participant is active in the interactions more than observing.
				A conversation among virtual actors (as was done for Phase 1 in the ICU) could model impaired dynamics at the team/organizational levels
12	3.50	1.38	Revisit for Round 2	Disagree, this implies that moral distress/injury is caused by bad leadership in healthcare
				Not a demonstration but a story of the same
				Most of the time, this is also a key cause of moral distress
				This could be a realistic component, but I don’t think essential.
				Betrayal at all three levels, might want to consider personal/family impact
				Poor or lack of leadership may help increase distress
13	3.58	1.24	Revisit for Round 2	To reduce complexity we should minimize branching
				Yes, this would help with future real-life scenarios that participants may encounter
				If possible that would be great because it was a criticism by participants of the scenario from Phase 1
				Didactic and reflective
				Ideal but challenging. it supports the idea that in "moral dilemmas" there may not be a right answer
14	3.58	0.79	Keep	It is important to educate the user. At the same time, this should not bias the experience of the participant.
				It's important that they can label the experience. I would think that many would already be familiar with the concept
				Recognition of distress in general and moral distress in particular
				I think that depends on the purpose of the study ... we can understand distress without this
15	3.67	1.67	Revisit for Round 2	I assume that it is that they experience moral distress during the initial phase, with the intent being that the intervention is designed to mitigate the moral distress
				Requiring is too strong a term. We have to assume that the challenge left them with something and that something is what is being addressed in some way in the intervention
				This is also an important requirement of this project
				This is likely not important if the participant can reflect on “real” moral distress they have experienced in their practice.
				Participant is coming into the 10-minute intervention with hopefully “moral distress,” the intervention should help reduce it
16	3.67	1.15	Revisit for Round 2	Like a moral stressor at work and then they rate symptoms as a result? I would go with the residue of the challenge and last month’s symptoms
				Will there be too many moving parts?
				Empirical research methods and proposals for measuring stress-related physiological response (e.g., inflammation) that will characterize the effects of population-level prolonged stress exposure on brain structure and function and on patterns of physiological response
				Helpful to see what we elicit
17	3.67	1.23	Revisit for Round 2	This all depends if we are trying to evoke other-directed MI or self-directed MI.
				Have someone share their story of mistakes and impact. Better yet, have a nurse tell a story of being overworked, having bad leadership and support, poor self-care, having patients and family members acting out their anger, and then at some point taking their anger out on patients and doing something they are ashamed of
				It should involve involving the participant also making mistakes, either intentionally or unintentionally
				If a mistake means doing something unethical then yes it is important. If a mistake is not present, then a problematic policy must be in the background--like limited staffing, discharging pts too early, etc.
				Making mistakes at individual/team/organizational levels--participants recognize this
				If the scenario is witness and transgression and decide to report
18	3.67	1.30	Revisit for Round 2	I don't have a strong opinion either way
				Not every moral distress scenario needs to involve a team, but it usually does
				Team dynamics might add an additional layer of complexity and might “mask” individual characteristics and trends
				The team aspect is essential because the relative power of nurses in the hospital hierarchy, along with its constraints, generally is at the center of moral distress experienced. The team should include administrators
				I feel moral dilemmas are quite personal and individual experiences
19	3.67	1.37	Revisit for Round 2	I think we should 86 this idea of helping people make better decisions. In morally complex and horrible situations there is no right way to respond that would make the experience not impactful and it is not the decision per se that is harmful but the situation and context. It is important to understand that moral injury is not only about doing harm to others but also personally transgressing, it is most commonly about bearing witness to injustice and bad acts or impossible moral stressors, or being the direct victim of other’s bad behaviors
				I believe it would be important to immerse participants in a decision-making situation in morally challenging contexts; however, another possible idea is to have them watch a discussion about an ethical dilemma or witness an unethical situation
				Decision-making is important for intervention, and the real-time aspect is a key component
				There must be something that constrains the participant from acting on their decisions.
				A decision tree might allow for a breakdown of decisions at individual/team/organizational levels, asking stress-related questions at each point of the decision tree and promoting reflective learning. the tree may be part of the education...
20	3.75	1.48	Revisit for Round 2	Not in real-time. The question should be: “What about the virtual experience resonates with you and how does it make you feel? And, how common is this experience, and does it interfere with the quality of your life and your functioning at work, home, and in terms of leisure?,” etc.
				A key goal is to make participants aware of being in a moral dilemma
				Again--moral dilemmas are distinct. They are about having two options that are equal but only one can be acted on so that the person doesn't know what to do. Moral distress is more about knowing what to do but not being able to act
				Coming out of a funeral in mourning would hopefully put the participant in a “stressful but reflective state”
				That should be the training. Likely a bridge too far for everyone
21	3.75	0.62	Keep	This would speak to the high-stakes situation
				unsafe settings with poor organizational oversight
				In health safety (health) is often the issue
22	3.83	1.11	Revisit for Round 2	Again here - it’s not clear if the intervention requires time-sensitive decisions, but rather the VR scenario incorporates time-sensitive decisions, as this will maximize the degree to which it mimics a scenario where moral distress is possible.
				Requiring is too strong a term. We have to assume that the challenge left them with something and that something is what is being addressed in some way in the intervention
				Time-sensitive decisions indicate stress and are commonplace in the healthcare setting
				Yes, they should make poor decisions in the moment
				Time is of the essence in all these
				It needs to be time-sensitive enough to be realistic, but again the distress isn't so much about the decision as the inability to act on one’s judgments/decisions
				Decision-making errors can be points for course correction
				I can’t see it being possible in 10 minutes without the pressure
23	4.00	0.74	Keep	This all depends if we are trying to evoke other-directed MI or self-directed MI
				Frustration isn’t necessarily a situation caused by a moral mistake
				This is possible in a natural scenario so good to include that component
				The participant needs to experience frustration in response to a constraint. All the actors do not need to be frustrated, however
				Frustration with maladaptive behavior at all levels
				I think frustration toward a participant may add to urgency and anxiety
24	4.08	1.24	Revisit for Round 2	This is the key feeling in moral distress
				It could be that nurses feel betrayed by the system.
				Betrayal at all 3 levels, might want to consider personal/family impact
				Yes they should react strongly regardless of the decision a participant makes
25	4.08	1.24	Revisit for Round 2	It is unrealistic that a healthcare professional would be working alone
				Moral distress usually involves more than just the person experiencing it. Someone else may have caused it
				Better than team dynamics as individual characteristics and trends may not be “masked”
				If the scenario is individualized it will be more realistic, but it will be more difficult to make conclusions across participants
				The individual is the focal point for reflection and bringing about change. Interaction with the group and organizational levels is important but it is individuals who are participating/reflecting and not groups/organizations
				I feel they are highly personalized and individual (almost lonely)
26	4.17	0.72	Keep	If someone is telling their story, maybe the thing could pause and the nurse could respond out loud. That could be good
				This could help with engagement in the scenario
				Interaction can bring more realism
				Interpersonal tension would be great
27	4.17	0.83	Keep	This is probably one of the main issues facing frontline HCW, and definitely needs to be embedded in the VR scenario design process
				Yes--this is a constant even if it is the background.
				Staffing issues could happen at anytime including COVID-19
				May be an issue if that creates pressure regarding a decision
28	4.17	0.94	Keep	I think to maximize response, the situation needs to be immersive and evocative
				I think the best challenge experience is to hear a very real and very compelling story of various moral harms and their impact
				Storytelling might divert the user from the actual medical event
				A generalizable scenario with an adverse event (e.g., death by suicide), having adverse events to both patient and nurse and how things are handled on an individual/team/organizational level might allow for evocative storytelling
29	4.25	0.87	Keep	The scenario needs to be able to evoke strong emotions - in fact, I would go so far as to exclude participants from analysis of the effect of the intervention if they do not exhibit some degree of distress during the initial scenario
				This is ideal but we have to find a way to be impactful when the scene is not very evocative. Many nurses will need to imagine these challenges and think about the future rather than having the capacity to be triggered because of personal moral stress
				I think this is where personalized aspects of the participant could be recorded and analyzed to provide specific parameters
				Important for objective and subjective data capture
				I would not call the sense of urgency and angst emotional
30	4.33	0.78	Keep	This is hard to pull off because you would need a benchmark for sufficient arousal to warrant concern. I think it is easier to simply ask folks the thoughts and feelings that come to mind as a result of the challenge and go from there. It would be important to correlate active and passive data for searching for potential physiological markers of stress/moral dilemma
				Diagnostic criteria reflecting the cluster of features and symptoms of “moral injury” that may be linked to but separate from PTSD can be used to accurately identify those affected Empirical research methods and proposals for measuring stress-related physiological response (e.g., inflammation) that will characterize the effects of population-level prolonged stress exposure on brain structure and function and on patterns of physiological response
				Biometric data captured in the VR setting should match the real-life ones.
				We are using distress as a surrogate for internal angst so the best we can do this
31	4.58	0.79	Keep	This will support learning and potentially lead to a bigger response
				Reflection could happen after the scenario is experienced
				I think the reflection could be helpful for learning
				This is the purpose of the scenario + intervention (compound intervention/Kolb’s model)
				I feel time pressure and urgency is key
32	4.58	1.16	Keep*	The situation needs to be something high stakes (e.g., life and death decision), trigger distress, have a moral component
				The situation needs to involve a difficult moral dilemma or moral decision that the nurse needs to make or observes someone else making
				It seems you are trying to replicate the idea of a moral dilemma and a nurse choosing a strategy as if the challenge should pertain to decision-making rather than an immersive experience that brings up moral emotions and personal experiences or worries about these things happening, which can then lead to a self-assessment and framing of the lasting impact of these experiences as moral harm moral stress and leaning about ways of managing and mitigating these very human and very understandable responses.
				Adverse outcomes as a result of individual/team/organizational decision-making with repercussions at the three levels. The adverse event could include the death of the patient (including by suicide) with a wrongful implication of the team/nurse and adverse outcomes to the nurse (could range from loss of job, reputation, maladaptive coping including addictions/drug use, or at the extreme stress/PTSD/depression/suicide, etc), the scenario could end at the funeral with participant exiting with PTSD
				Diagnostic criteria reflecting the cluster of features and symptoms of “moral injury” that may be linked to but separate from PTSD that can be used to accurately identify those affected;" better that participant is engaged as opposed to an observer in my opinion
33	4.67	0.65	Keep	Plan for the lowest common denominator which may be a minimal real-time emotional reaction
				This is would be a key component of the interventional strategy and any associated physiological and emotive responses and parameters
				Yes, but to be ethical the genuine reaction must be a small dose
				Maybe pertinent to get participants to name the “thought” or “emotion.” However, given that the scenario is only 10 minutes, this would have to be laid out as choices and not open ended
				The reaction may be internal distress but a decision or reaction/action is a must
34	N/A	N/A	N/A	Inpatient ward Community care ICU ER LTC Any unit General medicine
35	N/A	N/A	N/A	Again, I think the assessment should be based on theory - if the goal of the intervention is to reduce moral distress - then this is what should be the focus. The question then becomes how to measure moral distress - biometric markers could potentially be used to measure correlates of hypothesized moral emotions such as guilt, shame, and anger [I’m thinking simple things like arousal, though this one is non-specific]. Since we know very little about peri-situational reactions to moral distress, it is difficult to firmly provide hypotheses about what might be happening - but if I was to hazard a guess I would think we’d want to focus on self-reported emotional response; beliefs about the situation and the decision and potentially about the causality of the decision, etc. [I have a tool we’ve used to look at reactions to potentially morally injurious events that I am happy to share]
				I would administer a mood scale like the Positive and Negative Affect Scale referenced to the reaction to the challenge and at some point have them fill out the MIOS with respect to their own worst and most currently distressing potentially morally injurious nursing experience and based on that score provide feedback and suggestions
				Make use of qualitative approaches as much as possible
				The psychological trauma associated with moral injuries can lead to insomnia, depression, physical and psychological pain, and maladaptive behaviors, including isolation from friends and family, self-medication with alcohol and drugs, etc. While these symptoms are often ascribed to operational stress injuries, notably, PTSD, moral injuries produce “scars” that are not well captured by these current conceptualizations
				The means to identify events and circumstances that have the potential to cause “moral injury,” and to measure their severity, in First Responders and primary healthcare workers
				Does the data collected through app/wearables (pre-participation) determine stress levels at VR participation--after VR participation
				What subjective data collection and subjective-objective correlation are needed for model development? What baseline data can be collected while keeping REB constraints (e.g., mental health data-personal health information of nursing professionals)
36	N/A	N/A	N/A	Overall, I think the intervention should be grounded in theory about potential ways to mitigate, reduce or prevent moral distress
				Goals: to increase awareness of moral dilemmas; and to increase moral resilience, if possible
				The purpose of this challenge is to stimulate empirical research on “moral injury/distress” among healthcare workers and First Responders. It is hoped results will also act as a proxy for understanding the ways military personnel are likely to respond when faced with similar morally injurious circumstances during combat, peacekeeping operations or when responding to a future pandemic.
				“Evidence-based methods for the prevention of moral injuries and/or treatment therapies”
				Consider a didactic 10-minute version versus an interactive 10-minute version with a therapist. Didactive will cover more material but does not need to be a VR intervention
				Pre-reading before the scenario but this could bias responses as some may read/not read/assimilate
37	N/A	N/A	N/A	I think that the most important part of the scenario is to decide if it’s self or other-directed moral situation, and to set the situation up such that the participant either observes or has to make a high-stakes moral decision. To evoke moral distress, I also think it's important that it's highly interactive, to promote emersion in the situation, and to maximize the likelihood of the participant responding emotionally.
				GUILT & SHAME, ANXIETY, DISTRESS, AND BURNOUT ARE KEY
				While the risk of “moral injury” is typically associated with warfare and conflict, evidence from the frontline of the COVID-19 pandemic suggests that healthcare workers and First Responders are also suffering extreme psychological, cognitive, and emotional responses, including guilt and shame. This state of anxiety and distress is often described as burnout. However, the cluster of features and symptoms of moral injury is not adequately captured by interventions and treatments associated with burnout
				QUICK DECISION MAKING (TIME/RESOURCE CONSTRAINTS)-->REAL-TIME INSIGHT FOR DECISION MAKING-->CUTTING EDGE PREVENTION MODELS & TREATMENT STRATEGIES TO PROVIDE FOR LONG-TERM MENTAL HEALTH
				Canadian Armed Forces members operate in extremely difficult and dangerous operational contexts and situations, in which they routinely face complex moral and ethical dilemmas. All military members are trained to make quick moral-ethical decisions, at times, with only limited information. Still, some operational experiences can be profoundly distressing. These experiences can give rise to feelings of guilt and shame, which can be morally injurious and result in long-lasting mental health challenges and impairment if left unresolved
				time pressure, tragic moral dilemma
38	N/A	N/A	N/A	In general, I think all three components are key, and all three components should be driven by a theoretical model of moral distress/moral injury development and prevention
				I believe the VR scenario should be as realistic as possible and evoke a common situation nursing professionals must face, so participants can emotionally resonate with the virtual avatars and easily recall past experiences of moral dilemmas
				VR instrumentation comfort level is a key element as many may not have experience with headsets so ease of device usage should be factored in to avoid any device-related bias of interaction/bias during the actual VR experiment
				“SUMMARIZING KEY TASKS FROM THE DND CALL.” The goal is to understand the circumstances and events that can give rise to “moral injury/distress,” diagnostic criteria, prevention models, and treatment strategies (e.g., psychological, neurological, and endocrine interventions for prolonged stress-related response treatment).
				Evocative scenario with “truly” adverse outcomes to the patient & participant in scenario-->stress/moral distress-->DSM-based symptoms-particularly PTSD & differentiation from moral distress-->identifying factors/correlation between virtual & real life correlation of stress response-->measuring stress-related responses-development of predictive models--> reflective methods for prevention/treatment

**Table 4:** List of questions for modified DM Round 2. VR: virtual reality; DM: Delphi methodology

Questions #	Question
1	How important is it that the VR scenario is developed to: Have participants experiencing anger?
2	How important is it that the VR scenario is developed to: Have the participants identify their values in potential conflict/difficult citation portrayed in the scenario?
3	How important is it that the VR scenario is developed to: Have the participants been exposed to a betrayal at a team level?
4	How important is it that the VR scenario is developed to: Have the participants navigate the scenario using a decision-making tree under a time crunch?
5	How important is it that the VR assessments are developed to: Require the participant, in real-time during the scenario, to complete “perceptual scales” (e.g., anxiety scales/stress index) such as pop-up in-scenario quizzes (provide results concurrently) based on standardized instruments? (please refer to the videos from DND1 for samples)
6	How important is it that the VR scenario is developed to: Have participants making mistakes unintentionally/intentionally?
7	How important is it that the VR scenario is developed to: Focus on the individual participant but the scenario is embedded within a team setting?
8	How important is it that the intervention is developed to: Explicitly make the participant aware that they are experiencing moral distress?
9	Please provide us with a sketch of a potential scenario and intervention that includes as many of features as possible

In Round 2 of the modified DM, 10 out of 13 participants rated the importance of the inclusion of the eight items from Round 1 in a survey questionnaire and provided sketches of potential VR simulation scenarios and in-VR interventions using free-text fields. As a result of the statistical analysis and review of the free-text field commentaries provided, four items were removed and four items were retained. Five VR simulation scenarios and in-VR intervention sketches were provided (Table 5). After Round 2 ended, a Zoom meeting with the participants resulted in retaining the decision to rank the VR simulation scenarios and in-VR intervention sketches in Round 3.

**Table 5:** List of modified DM Round 2 items, free-text field comments, means, and SDs. * Item originally “Revisit for Round 3” based on statistical analysis, however, was reclassified based on comments/end-of-round meeting discussions. SD: standard deviation; ICU: intensive care unit; MI: moral injury; DM: Delphi methodology; COVID-19: coronavirus disease 2019; PTSD: post-traumatic stress disorder

Question	Average	SD	Decision for modified DM Round 2	Comments
1	3.44	0.73	Remove	More than anger, with moral distress, it is usually shame and guilt
				Anger is one possible emotion but I don’t think it is absolutely essential
				Participants need to experience and identify the emotion such as anger
				Anger or frustration is good if we hope to create an element of “betrayal” but not all events will cause anger
				Anger is a moral emotion that arises from exposure to others’ transgressive and betraying behaviors. It is half the moral injury puzzle. Shame is the moral emotion that arises from one’s own transgressive behaviors. So, it depends on the focus of the challenging task
2	3.67	1.22	Keep*	If they can name their values, it may be helpful in coping with the conflict
				There likely are value conflicts embedded in any moral distress situation involving nurses, but not all participants will be able to articulate them because they are inherent in social structures that devalue care work and those who provide it. It's impossible to fully understand moral distress without an analytical lens that can capture the power
				Naming the emotion and reflecting on underlying causes is important
				They may not see it during the initial VR exposure but, eventually, this is important
3	3.56	0.88	Keep	Despite some thought on the topic, it is often the leadership that betrays what the team is able to do, causing distress
				I have mixed thoughts about this one. The betrayal is often at the level of the institution as opposed to the team
				Participants need to experience and identify the emotion (e.g., betrayal) and reflect on underlying causes including at the team level
				again possible depending on if the scenario is meant to evoke “anger” rather than guilt/shame ... one scenario is unlikely to evoke all three
				If we are going for betrayal and no personal transgressive behaviors, this seems ecologically important for nurses and something that would hit home
4	3.44	1.13	Keep*	Immediate action without being able to think it through can lead to moral distress and situations causing it
				Will be limited by the time we have to create the VR scenario
				Depends on the scenario
				Time crunch (scarcity of time/resources for decision-making) is key. However, this could be portrayed in the scenario, the decision-tree approach could be better used for the participant to identify the emotion and causes at individual/team/organizational levels. A 10-minute scenario will not be enough time to have a participant go through a detailed decision tree with various branch points AND also stop and reflect on the emotion/cause. A feasible alternative is to get the participant to reflect on what the person in the scenario was experiencing as the scenario plays out
				I feel this is a good way of increasing some stress/distress with
				I don’t like this contingency. This assumes that we want to create a challenging task that requires decision-making as if the enterprise will help them make the right decisions under stress. Moral injury is what happens when people are betrayed by their own behaviors or those of others and these things are not strategies or choice points but non-conscious and non-deliberate and non-deliberative
5	3.33	1.50	Remove*	Is this the best way to collect the data? If we’re embedding these into the VR it would reduce the amount of time they can actively participate in the scenario. Something to consider
				It’s a good idea but it might be necessary to pull out items that measure emotion as opposed to mood
				See the comment above. There may be elements of the perceived stress scale or even participants stopping to name the emotion/cause at different time points
				This may interfere with the process perhaps reflection afterward will capture
				This will provide an informed design of labeling for signal/scene analysis
				I would not do this because it gets in the way of being present for the challenging task. Ask afterward. Why is real-time assessment needed?
6	3.11	1.54	Remove*	It depends on what is meant by a “mistake.” A more likely scenario would include someone misusing their power in a way that does not ultimately serve a patient's good
				See the comment above - the person in the scenario makes decisions under a time/resource crunch which leads to negative outcomes. The participant then reflects on the experience of the person in the scenario
				best is “no right answer,” i.e., perception of wrong
				I don't think this is the way to go
7	4.33	0.50	Keep	Emergent situations, or situations likely to cause moral distress are usually handled by a team
				I think to be realistic for nursing, a team context is important
				The person in the scenario makes decisions under a time/resource crunch with individual/team/organizational aspects which leads to adverse outcomes at the individual/team/organizational levels. The participant then reflects on the experience of the person in the scenario
				I think MI is an individual process that can occur in an individual or group setting
8	3.11	1.17	Remove*	Not necessarily immediately, but if they do not recognize their distress as moral, perhaps some prompt might be in place to help them.
				In the long run, I think is beneficial for participants so that they can label it when they experience it again. The best intervention is to support participants in being assertive and making organizational change
				The person in the scenario should clearly experience stress in general and moral distress in particular. The participant puts himself/herself in the shoes of the person in the scenario and reflects on the process.
				I am not sure it will be explicit...they will feel distressed but may not see it as values in conflict
				Not in real-time. Do something immersive so that the person can be present for it and then ask them afterward what is coming up for them right now
9	N/A	N/A	N/A	1- Perhaps a nurse caring for many more patients than they normally do without backup. Admins get involved and tell them they just have to do the best they can and no help is forthcoming. The nurse night take shortcuts to try to keep up, then makes a serious med error and a patient is harmed - necessitating movement to a higher level of care, or a patient dies. Perhaps while all this is going on the nurse intentionally ignores a call bell for help when a patient falls. The nurse gets blamed by the admin for both mistakes
				2- “A nurse who works in long-term care/home care etc. shows up to their shift and is told they are working short and have an increased assignment as a result. At some point, there is a lack of resources (PPE, meds, or equipment due to supply chain issues). At some point, they have a patient who is declining (medically) and one who is very emotionally distraught- RN has to choose who to care for. At some point, they are given an important task (e.g., sterile dressing change) that requires two people- they don’t have a second person, so either can’t do the task, or it’s done not to best practices. At some point, they have a colleague who confides in them they don’t have the energy to do their job, and/or aren’t doing their full roles/responsibilities because they are so burnt out. OR a colleague goes on a rant (unprofessionally) about another patient because they are burnt out. Consider lumping multiple triggers for moral distress together to maximize effect”
				3- “10-minute scenario outline Participant becomes the “nurse in the scenario” with other avatars at individual/team/organizational levels. The first five minutes- Early discharge from the inpatient unit (or emergency unit) due to time/resource crunch-patient death in the ICU with difficult family/team discussion. Stop at a few time points to get participants to reflect on being the nurse in the scenario. The second five minutes- Consequences to the nurse at individual/team/organizational level, the nurse has negative outcomes (burnout) and job loss. Stop at a few time points to get participants to reflect on being the nurse in the scenario”
				4- I am not sure I have one in mind. Above is a bit confusing. If we want anger and “institutional betrayal” then an urgent need, decision made but not enough supply (i.e., blame the organization for failing)
				5- Nurses working on a busy inpatient unit, they are short-staffed this shift and the nurse is also training a newly hired new graduate nurse during this shift. The nurse is unable to meet his / her patient's needs (e.g., cannot respond to an emergency situation in a timely manner) or has to reallocate resources/make decisions under a time crunch or nurse makes a mistake or witnesses another nurse/team member makes a mistake with poor patient outcome (e.g., administers a wrong dose of narcotic and patient experiences respiratory arrest/death). The nurse knows the right thing to do but cannot do it because of the system/environment. The nurse is angry, feels betrayed by the organization and the system, and feels guilty that the new graduate nurses are being introduced into the profession this way. Both patient well-being and professional well-being are impacted.
				6- “First five minutes: Frontline HCW discussion in LTC with patient’s family with complex/morally challenging decisions under time pressure at individual/team/organizational level leading to patient death (anger, betrayal, loss, etc). Second five minutes: Copying of frontline HCW with depression and ending in self-injury. INTERVENTION: 10 minutes: Instructional in a conversational context”
				7- As many as what features?
				8- “SCENARIO: Hospital is understaffed and nurses must make decisions about who on the team works in what area/unit that day, and are forced to place nurses who are untrained or unfamiliar in some areas/units. This scenario could be rigged such that a bad outcome occurs regardless of the decisions of the nurse, resulting in harm to a patient and/or administrative/disciplinary action against one of the nurse’s team members due to the choices of the nurse. INTERVENTION: a brief (~5-10 minutes) didactic on moral distress and mindfulness, possibly also on power and control (e.g., we could pull talking points from Cognitive Processing Therapy for PTSD handouts). Then an ~10-minute mindfulness exercise emphasizing grounding and attentional control (e.g., similar to attention training), to help the nurse create the space to recognize their reaction and identify what they have the power to do in this context”

In Round 3 of the modified DM, nine out of 13 participants ranked the five VR simulation scenarios and in-VR intervention sketches provided in Round 2 on a Likert scale of one to five, one being the most likely candidate and five being the least likely candidate. To assist with the ranking, participants were presented with the 16 items that were retained from the previous DM rounds as elements to be integrated into the content of the final VR simulation (Table 6).

**Table 6:** List of items retained from modified DM Rounds 1 and 2. VR: virtual reality; DM: Delphi methodology

The scenario would
*educate the participant about stress in general and moral distress in particular, and have them be able to label the experience
*have participants actively interact with virtual actors
*have participants exposed to betrayal and reflect on underlying causes at a team level
*have participants reflect on their reactions to the scenario in relation to their values/beliefs
*educate the participant about stress/moral distress and be able to help the participant label the experience
*have the participant navigate a time-constrained scenario using a decision-making tree
*include evocative and immersive storytelling
*trigger genuine, emotional responses in the participant (small doses)
*show that something high stakes had happened with a moral component
*focus on the individual participant but be embedded within a team setting
The virtual actors, due to the scenario, would
*feel unsafe to speak
*experience frustration and display maladaptive behavior
*feel the effects of staffing issues
The intervention
*is didactic on recognizing at individual/team/organizational levels stress→moral distress→values in conflict
*includes some interactive components related to the scenario for active learning
The assessments would
*use participant biometric data (e.g., physiological markers of stress levels) gathered during the VR simulation for pre-post intervention stress and moral distress comparison
*get participants to feel/think/reflect on the experience at different time points in the scenario→intervention→repeated scenario and examine the pre-post effects of the intervention

All five VR simulation scenarios and in-VR intervention sketches were rated as somewhat candidates to use as the content for the final VR simulation (Table 7). After Round 3 ended, a Zoom meeting with the participants suggested combining some of the elements from each of the five VR simulation scenarios and in-VR intervention sketches to create the content for the final VR simulation. The general content of the VR simulation and in-VR intervention was decided to be the following: in the VR simulation, the participant is a novice nurse and due to staffing shortages, needs to choose between attending to two patients who are in critical, near-death conditions. As a result, one patient who is not cared for dies, and the participant is then reassured by a colleague which is delivered in the format of an intervention on ways to mitigate moral distress. This was converted into a VR simulation script for the scenario and an in-VR slide deck as the didactic intervention and a focus group interview to review these materials took place afterward.

**Table 7:** List of modified DM Round 3 sketches, free-text field comments, means, and SDs. SD: standard deviation; DM: Delphi methodology; ICU: intensive care unit; ED: emergency department

Scenario stem #	Ranking	SD	Scenario suggestions	Intervention suggestions
5	2.22	1.2	This seems more tailored for an admin or middle manager. If we want to get to “boots on the ground” nurse distress this scenario is unlikely to do that, especially if the participant is not in admin	I like the ideas for the intervention provided in this stem
			No suggestions	“Intervention suggestions are excellent and could be incorporated into the 10-minute set up as things which could be done in addition to reflection to lower stress - mindfulness, attentional control, psychological first aids. These could be 20-second exercises within the otherwise didactic intervention”
			A nurse making decisions about allocating other nurses sounds like a “charge nurse” role. This may not be relatable for as many participants as not all nurses take on the charge nurse role	I can’t comment, not an expert in moral distress
			I like this scenario because it is realistic/relatable regarding the difficult and morally conflicting task of making staffing re-allocation decisions (redeployment) which occurred during several waves of the pandemic. I would suggest the nurse in the scenario is the charge nurse (or nurse manager). The bedside nurse does not typically make these decisions though a bedside nurse participant would be able to relate as they may have been redeployed themselves, or may know colleagues who were redeployed	I have no suggestions for the presented intervention
			Scenario would need expansion	
			It would be very difficult for a group of non-RNs to rig the system in a realistic way enough to get to the error. It could unintentionally lead the RN to think “well I would never do that” and we wouldn’t get the response we are looking for	
			This is a well-articulated scenario! Hope other scenarios especially #3 and #4 can provide this level of specificity	
			Good - forced to do something that compromises the value of the supervisor send only those prepared. Also, harm to the patient	
			Have some of the nurses complain about being assigned to unfamiliar areas/units, and have the participant pull rank and force them to work anyway	
4	2.44	1.33	I think this is a good scenario and introduces moral distress. This is a scenario that is played out in a big way recently. It needs more specifics - such as what is the emergency situation, what are decisions which need to be made, etc.	I would echo here the answer for scenario #1
			Vague. The least favoite option for this reason	This is not my area of expertise so I will not comment on ideas for an intervention
			“Perhaps combine this with some of the ideas in scenario 1: med error and/or fall. Adding a student nurse is an interesting idea. I wonder if/how that contributes to moral distress. Is it a value-add?”	The 10-minute intervention could be didactic on individual/team/organizational aspects with some non-didactic material tailored to the scenario. The most the intervention could achieve in the immediate term is to get people to reflect on their values/thoughts/perspectives/emotions
			I like this scenario for the less overt examples of mistakes and for acknowledging the current and ongoing context of learners (new graduate nurses, nursing students) in the environment and how that can play a part regarding the emotions triggered - I wrote this scenario so I am not sure if I was supposed to rank it	I can't comment, not an expert in moral distress
			Some pertinent elements but may need to be made more "challenging"	Basically aware that they are in a difficult situation and what values are in conflict and a possible process to make these decisions. forced to prioritize
			Like #2 is very realistic. It is also very distressing as relatively recently an RN in the US made a medication error that they were held liable for, so it's on the top of folks' radar and may trigger more of a response than otherwise	Explain how the participant could reach out for support about the organization’s “betrayal.” and how to guide the new graduate nurses
			The factors that constrain the nurse to perform their obligations could be made specific	
			Good overall lots in 10 minutes	
			Have the new graduate nurse ask questions and provide comments, like “shouldn't we report that?” when they notice that the other nurse/team member made a mistake	
2	2.77	1.2	This scenario is messy because it contains things that might cause distress, but not necessarily moral distress. I would axe this one. Long-term care is a huge issue and should be addressed, but if we are focusing on moral distress, this one does not do the job	This is not my area of expertise so I will not comment on ideas for an intervention
			No suggestions - I like the idea of multiple triggers to maximize effect, but could introduce unwanted/uncontrolled variability	The 10-minute intervention could be didactic on individual/team/organizational aspects with some non-didactic material tailored to the scenario. The most the intervention could achieve in the immediate term is to get people to reflect on their values/thoughts/perspectives/emotions
			I wonder how distressing weighing medical vs. emotional needs would be. I suspect most nurses would be somewhat accustomed to prioritizing medical needs	I can’t comment, not an expert in moral distress
			“I like this scenario for the realistic patients (medically unstable and emotionally distraught) and realistic nursing interventions and environment. This may not be applicable to a broad group of nursing participants due to the environment (long-term care/home care) but is still relevant/relatable. Of note, there are Registered Nurses (RNs) and Registered Practical Nurses (RPNs) at Unity Health Toronto, so please state “nurse” instead of “RN” in the scenarios - to be inclusive and representative of both types of nurse participants”	Explain to the participants that they have done all they could within their capabilities, and how to properly communicate/deal with a colleague that is making unprofessional remarks
			Perhaps 1 & 2 could be combined, the scenario may not be challenging enough	
			This is very realistic as many HCP rely on their colleagues to support them emotionally. It would also be fairly straightforward to design	
			This is an interesting scenario and appears to be a “busy one” and the main challenge would be to succinctly communicate this within the available time	
			Reasonable to make a difficult choice. this is more classic ie there is no right choice and either choice will lead to a negative outcome	
			At the end of the scenario, show what happened to the other patient that didn't receive service/care. Maybe show that a family member came to complain about that.	
1	3.11	1.53	This scenario contains many of the elements of moral distress and betrayal. However, it does need an intervention to assist the nurse in dealing with feelings. The problem is that everyone will react differently - some are more resilient already than others, but having a participant correctly label these feelings would tailor an intervention to assist them in sorting out the feelings and effectively dealing with them. There really is no “one size fits all” intervention. Betrayal of trust can have long-term effects that a short five-minute intervention cannot manage	We need to pick one intervention that has evidence behind it to use to determine its efficacy
			I think the concept and stress of working short-staffed is very realistic and relatable across most areas of healthcare/nursing right now	Walk through ethical decision-making; exploration of all the factors that contribute to the event
			This is not realistic regarding the “blame” component of this scenario. It is not that overt in a real situation, and in my experience, it is usually self-inflicted blame, vs. being blamed by “admins” (who are the “admins”?) Also, the leadership and teams in real situations are supportive to offer help or reallocate resources so help can be available	This is not my area of expertise so I will not comment on ideas for an intervention
			There could be “added” stress with individual/team/organizational impact (practice review, audits, supervised practice, reporting to the professional organization, etc.). At different time points, participant's perceptions could be gauged as done in Phase 1	The 10-minute intervention could be didactic on individual/team/organizational aspects with some non-didactic material tailored to the scenario. The most the intervention could achireve in the immediate term is to get people to reflect on their values/thoughts/perspectives/emotions
			This scenario feels very similar to stem # 2 and the latter already has more of the components we are looking for. Suggest stem #2	I can’t comment, not an expert in moral distress
			The intensity of the scenario could be increased by assuming the patient has died rather than escalating to a higher level of care. Also, the situation regarding patient falls could be expanded by providing a resultant serious medical condition as an example after the fall	just ensure people are aware of the values in conflict ie leadership should be supportive and help me do my job to the best of my abilities
			Good although it appears are setting the scenario up as an “institutional betrayal” rather than a direct struggle with values .. i.e., my leadership has put me in an impossible situation	Present the support mechanisms that the nurse can rely on if he/she is under pressure from the admin
			Nurse is sick but is forced to go to work because it doesn’t have paid sick days or is forced by the admin, and a patient ends up getting sick	
3	3.77	1.2	ED and ICU are completely different, though they do impact each other, this one also is not realistic with too much in here. The ED nurse would not be dealing with family in the ICU	This is not my area of expertise so I will not comment on ideas for an intervention
			I think this stem is less clear than options 1 & 2	The 10-minute intervention could be didactic on individual/team/organizational aspects with some non-didactic material tailored to the scenario. The most the intervention could achieve in the immediate term is to get people to reflect on their values/thoughts/perspectives/emotions
			Is it realistic for the negative outcomes (burnout, job loss) to be so obvious and immediate after only one event?	I can’t comment, not an expert in moral distress
			Scenario outline looks good, but needs more color & drama!	As above basically aware that they are in a difficult situation and what values are in conflict and a possible process to make these decisions
			This would be challenging as it would have almost infinite branching	Explain how the participant could get support
			The aspect of a job loss should be clarified as to whether it is a resignation due to burnout or a layoff	
			Unclear if the nurse is a passive witness. I think better if engaged	
			Make it feel as if the patient’s death was blamed on the participant (maybe during the family discussion)	

Feedback from the focus group interview

The feedback provided after the focus group interview led to seven additional rounds of iterations to both the VR simulation script and slide deck via email. Main changes to the VR simulation script included (1) removing the racialized components (i.e., racialized names and accents) to avoid possibly reinforcing stereotypes, (2) making the narration and speech of the virtual characters more natural and realistic (e.g., instead of “what kind of professional are you?!,” the text was changed to “what’s wrong with you?!”), and (3) incorporating rage in the decision tree options for participants to pick when prompted (Table 8). Concerning the major updates to the in-VR intervention slide deck, they involved: (1) removing transitional or introductory slides that led from one topic into the next to run the intervention within the time constraints of the VR simulation (i.e., within 10 minutes), and (2) rephrasing the symptoms of moral distress and strategies to mitigate moral distress to better convey the learnings to the end user (Table 9). The VR simulation prototype was also improved to (1) include more facial and verbal expressions in the virtual characters to provoke emotional reactions in end users, (2) add natural body movements to the virtual characters (previously, the animation was set to idle so virtual characters were looking down and swayed side to side) to increase realism, and (3) have a professional voice actor narrate. All of these changes were made with the intent to align with the objectives of the educational resource to understand and mitigate moral distress in healthcare workers (Video 1).

**Table 8:** Feedback provided from the focus group interview on the VR simulation script. VR: virtual reality

Version	Updates
1	N/A
2	Added MIOS and IPQ scales
3	Changed Patient #2’s surname to avoid stereotypes; Changed Patient #2’s diagnosis from eclampsia to stroke; Skin tone as a randomized feature; Removed patient accents; Renamed RPN to “nursing colleague”; Removed nursing colleague’s reference to feeling burnt out; Added more decision trees (DTs); Removed reference to Patient 1 being an important person and receiving preferential treatment; Added outgoing nurse performing the Transfer of accountability; Added Code Blue being called by the ward admin; Changed Patient #2’s husband to be less punitive and more empathetic; Specified in the script indications of actions and narration
4	Added answers with rage to the DTs; Remove Patient #2’s husband accusing the staff of thinking Patient #2 is hysterical
5	Added tutorial to the VR scenario; Updated actions and narration. Some actions were changed to be narrated passages; Updated MIOS questions; Added details of the location for each passage; Removed the threat of the ward admin to report to the hospital director
6	Added more narrated elements. Additional actions were replaced by narration; improved clarity of description of actions; Changed narrator referring to the participant from “participant” to “you”; Added a new character (doctor) to show support and empathy to the participant and the end of the story; Added in the tutorial a reference to unburdening to be done by a virtual avatar
7	Changed skin tones from randomized to preset; Removed other demographics features apart from gender and skin tone; Removed from tutorial a reference to unburdening to be done by a virtual avatar; Minor updates to a few dialogues to sound more natural

**Table 9:** Feedback provided from the focus group interview on the in-VR intervention slide deck. VR: virtual reality

Version	Updates
1	N/A
2	Decreased size from 40 to 29 slides; Added reference to COVID-19 in the problem statement of MD in healthcare; Added content about stress, moral distress (MD), and the difference between both; Removed content about moral emotion, the difference between MD and moral injury (MI); Removed slide about takeaways from the intervention; Added content about physiological, emotional and cognitive responses to stress; Removed content related to MI; Removed MD related to COVID-19 restrictions and measures; Added more interventions to mitigate MD: breathing exercises; Removed recommendations and rationale for a nurse leader to be a support team member; Slight aesthetic improvements
3	Decreased slide count to 18; Reduced content related to interventions at the individual, team, and organizational levels; Removed all specific interventions to mitigate MD
4	Removed problem statement; Added a slide with a summary of the intervention slide set; Reduced content on stress (physiological, cognitive, and emotional responses) and MD; Added more interventions to mitigate MD: grounding techniques (diaphragmatic breathing, 5-4-3-2-1 technique, self-compassion); Added links to YouTube videos as examples of each intervention
5	Changed slide format to script; Further shortened content
6	Improved accuracy of the content on MD in healthcare, symptoms of MD, strategies to mitigate MD, and specific interventions to mitigate MD
7	Changed format back to slide set and (slide count:15); General decrease in content (which was reorganized across the slides); Removed the summary slide added in the previous version; Removed slide about moral stressors; Removed YouTube links; Added a new intervention: unburdening; Style improvements (added avatars while considering diversity; added smooth slide transitions)
8	Changed format to video; Reduced content on symptoms of MD; Removed the 5-4-3-2-1 grounding technique; Improved content on the unburdening intervention; Slight improvements to style and text accuracy

**Video 1:** Video recording of the first VR simulation prototype that resulted from the CIC approach. VR: virtual reality; CIC: constraints-ideation-consensus

SWOT analysis on the constraints-ideation-consensus approach

Participants who took part in the CIC approach were sent a Google Form survey in the form of a SWOT analysis to provide feedback on the methodology. The SWOT analysis is a technique for identifying and analyzing internal and external opportunities and threats [23].

In terms of strengths, participants stated that the approach was an excellent way to capitalize on and integrate expert knowledge of a wide range of areas of expertise relatively efficiently. They felt that it organized thought processes well and that it offered an equitable way of choosing key content areas for the development of the VR simulation. Lastly, they noted that it gave them an opportunity for everyone to speak out their ideas and suggestions that otherwise would not be feasible in large and hierarchical group discussions. 

With regards to weakness, participants mentioned that it was challenging for them to commit and follow through all stages of the process as it spanned for around two months. During this time participants were required to attend a two-hour DT session, answer three rounds of DM survey questionnaires, and attend focus group interviews after each round. They felt that this process was time-consuming and long, and they were not sure if they were present or engaged throughout. Particularly for the DM rounds, there were concerns with how the responses from individuals were weighted. As there were people from various backgrounds, those with no background in moral injuries, such as simulation experts, had their opinion count equally as those with a background in moral injuries, such as psychologists and nurses, which could have led to watering down the responses of the different experts input provided through the DM. Another pain point of the CIC approach was that participants felt that sometimes it resulted in content not grounded in theory. For example, the rationale for the interventions was somewhat post hoc - they seemed to be chosen primarily because they were a good fit for the study design not because of any theoretical connection with target processes. Participants also thought that the large size of the group may have slowed down the efficiency of the process.

The opportunities that the CIC approach yielded for participants was that it was able to offer fresh perspectives and more clarity on moral distress. The virtual setting used meant a wide range of experts from multiple locations could regularly interact and collaborate. Finally, the CIC approach allowed for the project to be constrained by an initial methodology and understanding of assessing and mitigating moral injury in healthcare workers which guided the brainstorming and discussions on the topic. 

The threats that were imposed on the CIC approach included participants’ limited availability to meet, low engagement, and not being able to follow through the steps as planned. Also, while online interactions allowed geographically distant participants to join, they lacked the same effectiveness as physical meetings where ideation and paper prototyping can be jointly done. Tight timelines of the project which did not allow for enough information shared far enough in advance were also noted to have a negative impact on the process by participants. Overall, through this SWOT analysis survey, participants provided valuable insights into the novel CIC approach.

## Discussion

This original article tested a novel CIC approach to describe the generation of parameters, needs, and requirements for the development of content for a VR simulation as a part of an educational resource to understand and mitigate the effects of moral distress on healthcare workers. To accomplish this, we utilized a unique expert-informant crowdsourcing method which consisted of a combination of constraints settings, DT and DM, followed by a focus group interview. The advantage of having employed DT for the development of content for the VR simulation is that it generated and expanded ideas in a creative and collaborative way [16]. However, this process alone lacks the rigor expected by the healthcare simulation community as the ideas lack evidence of content validity [25]. Therefore, we skip the final two phases of the DT to narrow down the ideas to a few executable ones using expert consensus through a DM before we prototyped and tested the VR simulation. The DM is widely accepted in the medical simulation community as a tool that provides content validity evidence [24]. The output of the DM was used to draft a scenario and intervention to embed into the VR simulation. An initial VR simulation prototype was created based on this output. To ensure that the VR simulation accurately conveyed the outputs, a focus group interview with the participants was conducted online.

With regard to the CIC approach, there were benefits relating to its execution and output. Starting the process by breaking down the problem into scenarios, interventions, and assessments for the VR simulation ensured that all aspects of the educational resource were being thought of and addressed by the participants. The way in which the CIC approach guided the participants in understanding what the needs for the content for the VR simulation are, garnering more clarity in every phase, was helpful. It prepared the participants to be able to offer suggestions on possible “stems” for VR simulation in the final phase of the CIC approach. This resulted in five possible stems which provided a base for the content for the VR simulation script and slide deck, fast-tracking the creation of a preliminary draft to feed into the VR simulation development process. The CIC approach also posed some challenges. For instance, during the DM, although a specific statistical algorithm was established to classify the items as “remove,” “keep,” or “revisit,” many of the comments did not correspond to this statistical algorithm. Specifically, many items were initially labeled as needing revision or clarification in the subsequent rounds of DM based on the algorithm. However, a closer look at the free-text fields linked to the items revealed various ideas were to be classified as either “remove” or “keep.” As a result, the analysis of the DM at the end of every round was difficult to conduct because both the statistics and the free-text comments needed to be examined. What alleviated some ambiguity around the interpretation of end-of-round DM results were the group meetings that occurred after each DM round where further discussion on these particular topics ensued.

The SWOT analysis completed by the participants also highlighted valuable insights into the CIC approach. Overall, participants felt that the process facilitated the discussion between various disciplines well, encouraged all viewpoints, and organized and refined the ideas in a logical and structured manner. The virtual platform enabled many individuals from various backgrounds situated across North America to participate. Mainly, it was the nature of DT and DM embedded within the CIC approach that allowed for the large group to work cohesively together [16,26]. A weakness and threat that was shared by the participants on the process was that it was long and over time they felt they became disengaged, thus not being able to contribute as much as they would have liked to. The online setting in which this process took place is mentioned in the literature to have this effect on participants due to the isolation and disconnection between them and the other participants [27]. What may have also led to mental withdrawal in participants could have been the widely contrasting opinions offered by the interdisciplinary group. To elaborate, the perspectives provided during the rounds for participants to review and rate as a part of the subsequent round may have become unmanageable and outside of the scope and interest of certain experts (e.g., details on moral distress provided by psychologists; however, computer scientists as one of the experts participating in the Delphi are expected to weigh in [28]. As considerations for future CIC approach use, the DT and post-DM rounds could be done as a group meeting in person in which all stakeholders could be seen physically (i.e., non-verbal communication) and viewpoints could be better articulated organically. To improve the exchange of ideas and knowledge between the different participating experts in the CIC approach, specific points that need input from a particular area (e.g., nursing) could be called to comment instead of having all expertise involved in the CIC approach expend their energy in trying to talk on items that they may not have knowledge on (e.g., simulationists being asked to address VR technical questions). The combination of a face-to-face setup along with being selective on how to use the participating stakeholder’s knowledge could allow for improved collaboration and outcomes from the CIC approach.

The use of online tools such as Zoom and Google Jamboards to facilitate the DT process and Google Forms to execute the DM allowed for the participants to share their ideas for the design of the VR simulation remotely and instantaneously, contributing to the speed of data collection [29,30]. The downside to Zoom is that it can pose technological problems (i.e., Internet connectivity and hardware issues related to audio) that can limit individuals from contributing their ideas. The disadvantage to Google Jamboards and Google Forms to collect data on the content for the VR simulation is that information provided by participants can be difficult to interpret and may require follow-up whereas in-person this could be resolved more readily. Despite these shortcomings, overall, the CIC approach was successful in delivering useful information from participants on the content for the VR simulation we aimed to present in this original article.

Lastly, a limitation that this original article presents is that although there is very little research on factors on and ways to mitigate moral distress, the in-VR intervention was based on “A guide to moral injury.” This was one of the few resources that were known to psychologists and psychiatrists on the team to provide some guidance on moral distress for the development of the educational resource and so, as a starting point, the decision was made to build upon this aid.

## Conclusions

The CIC approach demonstrates feasibility in leading to the identification of parameters, needs, and requirements for the development of an expert-validated VR simulation early prototype that, with testing, could be used as an educational resource for healthcare workers to understand and mitigate moral distress. The methodology also proved to be flexible by morphing ideas into questions about the content of the VR simulation that were able to collect critical, stakeholder-approved, elements for an educational resource. The applicability of this process for other healthcare simulations seems promising based on our findings; however, it requires further testing by the research community to fully explore its potential.
